# Summary of Guidance for Minimizing the Impact of COVID-19 on Individual Persons, Communities, and Health Care Systems — United States, August 2022

**DOI:** 10.15585/mmwr.mm7133e1

**Published:** 2022-08-19

**Authors:** Greta M. Massetti, Brendan R. Jackson, John T. Brooks, Cria G. Perrine, Erica Reott, Aron J. Hall, Debra Lubar, Ian T. Williams, Matthew D. Ritchey, Pragna Patel, Leandris C. Liburd, Barbara E. Mahon

**Affiliations:** 1 CDC COVID-19 Emergency Response Team.

As SARS-CoV-2, the virus that causes COVID-19, continues to circulate globally, high levels of vaccine- and infection-induced immunity and the availability of effective treatments and prevention tools have substantially reduced the risk for medically significant COVID-19 illness (severe acute illness and post–COVID-19 conditions) and associated hospitalization and death (*1*). These circumstances now allow public health efforts to minimize the individual and societal health impacts of COVID-19 by focusing on sustainable measures to further reduce medically significant illness as well as to minimize strain on the health care system, while reducing barriers to social, educational, and economic activity (*2*). Individual risk for medically significant COVID-19 depends on a person’s risk for exposure to SARS-CoV-2 and their risk for developing severe illness if infected (*3*). Exposure risk can be mitigated through nonpharmaceutical interventions, including improving ventilation, use of masks or respirators indoors, and testing (*4*). The risk for medically significant illness increases with age, disability status, and underlying medical conditions but is considerably reduced by immunity derived from vaccination, previous infection, or both, as well as timely access to effective biomedical prevention measures and treatments (*3*,*5*). CDC’s public health recommendations change in response to evolving science, the availability of biomedical and public health tools, and changes in context, such as levels of immunity in the population and currently circulating variants. CDC recommends a strategic approach to minimizing the impact of COVID-19 on health and society that relies on vaccination and therapeutics to prevent severe illness; use of multicomponent prevention measures where feasible; and particular emphasis on protecting persons at high risk for severe illness. Efforts to expand access to vaccination and therapeutics, including the use of preexposure prophylaxis for persons who are immunocompromised, antiviral agents, and therapeutic monoclonal antibodies, should be intensified to reduce the risk for medically significant illness and death. Efforts to protect persons at high risk for severe illness must ensure that all persons have access to information to understand their individual risk, as well as efficient and equitable access to vaccination, therapeutics, testing, and other prevention measures. Current priorities for preventing medically significant illness should focus on ensuring that persons 1) understand their risk, 2) take steps to protect themselves and others through vaccines, therapeutics, and nonpharmaceutical interventions when needed, 3) receive testing and wear masks if they have been exposed, and 4) receive testing if they are symptomatic, and isolate for ≥5 days if they are infected.

## Vaccines and Therapeutics To Reduce Medically Significant Illness

**COVID-19 vaccination.** COVID-19 vaccines are highly protective against severe illness and death and provide a lesser degree of protection against asymptomatic and mild infection (*6*). Receipt of a primary series alone, in the absence of being up to date with vaccination* through receipt of all recommended booster doses, provides minimal protection against infection and transmission (*3**,**6*). Being up to date with vaccination provides a transient period of increased protection against infection and transmission after the most recent dose, although protection can wane over time. The rates of COVID-19–associated hospitalization and death are substantially higher among unvaccinated adults than among those who are up to date with recommended COVID-19 vaccination, particularly adults aged ≥65 years (*5*,*7*). Emerging evidence suggests that vaccination before infection also provides some protection against post–COVID-19 conditions,^†^ and that vaccination among persons with post–COVID-19 conditions might help reduce their symptoms (*8*). Continuing to increase vaccination coverage and ensuring that persons are up to date with vaccination are essential to preventing severe outcomes. Overall booster dose coverage in the United States remains low,^§^ which is concerning given the meaningful reductions in risk for severe illness and death that booster doses provide and the importance of booster doses to counter waning of vaccine-induced immunity. Public health efforts to expand reach and promote equitable access to vaccination have resulted in similar rates of primary series coverage across most racial and ethnic groups (*9*); however, racial and ethnic disparities in booster coverage have emerged (*10*). Supporting community partnerships and leveraging trusted sources of information must continue in order to eliminate persistent disparities and achieve equity in booster dose coverage, including through increasing education efforts and promotion of equitable vaccination outreach. Public health efforts need to continue to promote up-to-date vaccination for everyone, especially with vaccines targeting emerging novel variants that might be more transmissible or immune-evasive.

**Preexposure prophylaxis.** COVID-19 vaccine effectiveness against severe outcomes is lower in persons who are immunocompromised than in those who are not, and persons who are immunocompromised and have COVID-19 are at increased risk for intensive care unit admission and death while hospitalized, irrespective of their vaccination status (*11*,*12*). Preexposure prophylaxis with Evusheld^¶^ can help protect persons with moderate to severe immunocompromise who might not mount an adequate immune response after COVID-19 vaccination, as well as persons for whom COVID-19 vaccination is not recommended because of their personal risk for severe adverse reactions. In addition to early antiviral treatment if infected, persons who are moderately or severely immunocompromised can benefit from COVID-19 preexposure prophylactic medication to help prevent severe COVID-19 illness, as an adjunct to up-to-date vaccination for themselves and their close contacts, early testing, nonpharmaceutical interventions, and prompt access to treatment if they are infected.

**Medications to treat COVID-19.** Antiviral medications (Lagevrio [molnupiravir], Paxlovid [nirmatrelvir and ritonavir], and Veklury [remdesivir]) and monoclonal antibodies (bebtelovimab) are available to treat COVID-19 in persons who are at increased risk for severe illness,** including older adults, unvaccinated persons, and those with certain medical conditions^††^ (*13*). Antiviral agents reduce risk for hospitalization and death when administered soon after diagnosis. The federal Test to Treat initiative facilitates rapid, no-cost access to oral COVID-19 treatment for eligible persons who receive a positive SARS-CoV-2 test result.^§§^ Recent expansion of prescribing authority of Paxlovid to pharmacists intends to further facilitate access.^¶¶^ Continued efforts are needed to reduce racial and ethnic differences in receipt of monoclonal antibody therapies (*14*) and disparities in dispensing rates for oral antiviral prescriptions by community social vulnerability (*15*).

## COVID-19 Prevention Strategies

**Monitoring COVID-19 Community Levels to guide COVID-19 prevention efforts.** Persons can use information about the current level of COVID-19 impact on their community to decide which prevention behaviors to use and when (at all times or at specific times), based on their own risk for severe illness and that of members of their household, their risk tolerance, and setting-specific factors. CDC’s COVID-19 Community Levels reflect the current effect of COVID-19 on communities and identify geographic areas that might experience increases in severe COVID-19–related outcomes, based on hospitalization rates, hospital bed occupancy, and COVID-19 incidence during the preceding period*** (*1*). Prevention recommendations based on COVID-19 Community Levels have the explicit goals of reducing medically significant illness and limiting strain on the health care system. At all COVID-19 Community Levels (low, medium, and high), recommendations emphasize staying up to date with vaccination, improving ventilation, testing persons who are symptomatic and those who have been exposed, and isolating infected persons. At the medium COVID-19 Community Level, recommended strategies include adding protections for persons who are at high risk for severe illness (e.g., use of masks or respirators that provide a higher level of wearer protection). At the high COVID-19 Community Level, additional recommendations focus on all persons wearing masks indoors in public and further increasing protection to populations at high risk.^†††^ As SARS-CoV-2 continues to circulate, changes in COVID-19 Community Levels for a jurisdiction help signal when use of some prevention strategies should be discontinued or increased, based on an individual person’s level of risk for severe illness or that of their household or social contacts. The COVID-19 Community Levels provide a broad framework for public health officials and jurisdictions to use and adapt as needed based on local context by combining local information to assess the need for public health interventions.

**Nonpharmaceutical interventions.** Implementation of multiple prevention strategies helps protect individual persons and communities from SARS-CoV-2 exposure and reduce risk for medically significant illness and death by reducing risk for infection (Table). Implementation of multiple nonpharmaceutical preventive interventions can complement use of vaccines and therapeutics, especially as COVID-19 Community Levels increase and among persons at high risk for severe illness. CDC’s COVID-19 prevention recommendations no longer differentiate based on a person’s vaccination status because breakthrough infections occur, though they are generally mild (*16*), and persons who have had COVID-19 but are not vaccinated have some degree of protection against severe illness from their previous infection (*17*). In addition to strategies recommended at all COVID-19 Community Levels, education and messaging to help individual persons understand their risk for medically significant illness complements recommendations for prevention strategies based on risk.

**TABLE:** Person- and community-level public health strategies to minimize the impact of COVID-19 on individual persons, communities, and health care systems — United States, August 2022

Recommended public health strategy	Person- and household-level prevention behaviors	Community-level prevention strategies*	Links to guidance and scientific evidence
COVID-19 vaccination	Stay up to date with COVID-19 vaccination	Distribute and administer vaccines to achieve high community vaccination coverage and ensure health equity Support community partnerships and leverage trusted sources of information to expand booster coverage	Vaccines for COVID-19: https://www.cdc.gov/coronavirus/2019-ncov/vaccines/index.html Stay up to date with COVID-19 vaccines: https://www.cdc.gov/coronavirus/2019-ncov/vaccines/stay-up-to-date.html Science brief: COVID-19 vaccines and vaccination: https://www.cdc.gov/coronavirus/2019-ncov/science/science-briefs/fully-vaccinated-people.html
Preexposure prophylaxis	Persons who are moderately or severely immunocompromised might benefit from COVID-19 preexposure prophylactic treatment (Evusheld) to prevent severe COVID-19 illness	Provide education and communication outreach to patients and clinical care organizations that serve patients with immunocompromising conditions to support equitable access to preexposure prophylaxis	COVID-19 preventive medication: https://www.cdc.gov/coronavirus/2019-ncov/need-extra-precautions/people-with-medical-conditions.html#preventive Prevention of SARS-CoV-2 infection: https://www.covid19treatmentguidelines.nih.gov/overview/prevention-of-sars-cov-2/
Medications for treatment of COVID-19	Persons at increased risk for severe illness should have a plan for rapid access to tests and treatment if they become infected	Enable rapid access to oral COVID-19 treatment within ≤5 days of diagnosis Support clinical-community linkages to ensure access to antiviral and monoclonal antibody treatment and reduce health disparities	COVID-19 treatments and medication: https://www.cdc.gov/coronavirus/2019-ncov/your-health/treatments-for-severe-illness.html Clinical management of COVID-19: https://www.covid19treatmentguidelines.nih.gov/management/clinical-management/
Improved ventilation	Increase ventilation and filtration	Take steps to increase ventilation and filtration in public places	Improving ventilation in your home: https://www.cdc.gov/coronavirus/2019-ncov/prevent-getting-sick/Improving-Ventilation-Home.html Ventilation in buildings: https://www.cdc.gov/coronavirus/2019-ncov/community/ventilation.html Ventilation in schools and childcare programs: https://www.cdc.gov/coronavirus/2019-ncov/community/schools-childcare/ventilation.html Science brief: SARS-CoV-2 transmission: https://www.cdc.gov/coronavirus/2019-ncov/science/science-briefs/sars-cov-2-transmission.html
Masks and respirators	Persons at high risk for severe illness should wear a mask or respirator (N95/KN95) that provides more protection indoors in public at medium and high COVID-19 community levels All persons should wear well-fitting masks or respirators indoors in public at high COVID-19 Community Levels^†^	Recommend all persons wear well-fitting masks or respirators at high COVID-19 Community Levels and support use of masks through messaging and resources	Masks and respirators: https://www.cdc.gov/coronavirus/2019-ncov/prevent-getting-sick/types-of-masks.html Science brief: community use of masks to control and spread of SARS-CoV-2: https://www.cdc.gov/coronavirus/2019-ncov/science/science-briefs/masking-science-sars-cov2.html
Testing	Persons with a known or suspected exposure to someone with COVID-19 and those who experience symptoms should promptly seek testing through point-of-care and at-home tests	Increase equitable access to testing, including through point-of-care and at-home tests for all persons Recommend use of screening testing in certain high-risk settings (e.g., long-term care facilities or correctional facilities) to reduce risks of outbreaks Support Test to Treat and other initiatives to support rapid access to treatment among persons at high risk for severe illness	Overview of testing for SARS-CoV-2: https://www.cdc.gov/coronavirus/2019-ncov/hcp/testing-overview.html Technical page: guidance for healthcare workers about COVID-19 (SARS-CoV-2) testing: https://www.cdc.gov/coronavirus/2019-ncov/hcp/testing.html
Isolation	Symptomatic persons should isolate promptly and seek testing Infected persons should stay home for ≥5 days; for 10 days, infected persons should wear a mask around others at home and in public and avoid contact with persons at high risk for severe illness^¶^	Increase equitable access to testing, including through point-of-care and at-home tests for all persons Support case investigation and contact tracing in high-risk settings where recommended	Isolation: https://www.cdc.gov/coronavirus/2019-ncov/your-health/isolation.html Science brief: SARS-CoV-2 transmission: https://www.cdc.gov/coronavirus/2019-ncov/science/science-briefs/sars-cov-2-transmission.html
Managing exposures to SARS-CoV-2	Persons with recent exposure should wear a mask indoors in public for 10 days and test ≥5 days after last exposure	Increase equitable access to testing, including through point-of-care and at-home tests for all persons Support case investigation and contact tracing in high-risk settings where recommended^§^	What to do if you are exposed: https://www.cdc.gov/coronavirus/2019-ncov/your-health/if-you-were-exposed.html Definition of close contacts: https://www.cdc.gov/coronavirus/2019-ncov/php/contact-tracing/contact-tracing-plan/appendix.html#contact Science brief: SARS-CoV-2 transmission: https://www.cdc.gov/coronavirus/2019-ncov/science/science-briefs/sars-cov-2-transmission.html
Hand hygiene	Wash hands frequently	Ensure provision of adequate hand sanitation supplies	How to protect yourself and others: https://www.cdc.gov/coronavirus/2019-ncov/prevent-getting-sick/prevention.html Science brief: SARS-CoV-2 transmission: https://www.cdc.gov/coronavirus/2019-ncov/science/science-briefs/sars-cov-2-transmission.html
Increasing space and distance	Persons at high risk for severe illness can consider avoiding crowded areas and minimizing direct physical contact, especially in settings where there is high risk for exposure	Provide education to populations at high risk for severe illness to advise them to consider taking steps to protect themselves in settings where there is high risk for exposure	How to protect yourself and others: https://www.cdc.gov/coronavirus/2019-ncov/prevent-getting-sick/prevention.html Science brief: SARS-CoV-2 transmission: https://www.cdc.gov/coronavirus/2019-ncov/science/science-briefs/sars-cov-2-transmission.html

* Recommended strategies relate to general community settings; adapted setting-specific guidance and recommendations include schools and early childhood settings (https://www.cdc.gov/coronavirus/2019-ncov/community/schools-childcare/k-12-childcare-guidance.html), high-risk congregate settings such as correctional facilities and homeless shelters (https://www.cdc.gov/coronavirus/2019-ncov/community/high-risk-congregate-settings.html), health care settings (https://www.cdc.gov/coronavirus/2019-ncov/hcp/infection-control-recommendations.html), and travel (https://www.cdc.gov/coronavirus/2019-ncov/travelers/index.html).

^†^ Although all masks and respirators provide some level of protection, properly fitting respirators provide the highest level of protection. Persons may consider the situation and other factors when choosing a mask or respirator that offers greater protection. https://www.cdc.gov/coronavirus/2019-ncov/prevent-getting-sick/types-of-masks.html#DifferentSituations

^§^ Universal case investigation and contact tracing are not recommended for COVID-19; health departments and jurisdictions should prioritize investigation of COVID-19 cases, clusters, and outbreaks involving high-risk congregate settings such as long-term care facilities and correctional facilities or unusual clusters of cases. https://www.cdc.gov/coronavirus/2019-ncov/php/contact-tracing/contact-tracing-plan/prioritization.html

^¶^ Infected persons should end isolation only when they are without a fever for ≥24 hours without use of medication and all other symptoms have improved. Persons who had moderate illness from COVID-19, including those who show evidence of lower respiratory disease such as shortness of breath or difficulty breathing should isolate for ≥10 days. Persons who had severe illness from COVID-19 (including those who were hospitalized or required intensive care) and persons who are immunocompromised should consult with a health care provider about how to determine end of isolation. https://www.covid19treatmentguidelines.nih.gov/overview/clinical-spectrum/

**Testing for current infection.** Diagnostic testing can identify infections early so that infected persons can take action to reduce their risk for transmitting virus and receive treatment, if clinically indicated, to reduce their risk for severe illness and death. All persons should seek testing for active infection when they are symptomatic or if they have a known or suspected exposure to someone with COVID-19. When considering whether and where to implement screening testing of asymptomatic persons with no known exposure, public health officials might consider prioritizing high-risk congregate settings, such as long-term care facilities, homeless shelters, and correctional facilities, and workplace settings that include congregate housing with limited access to medical care.^§§§^ In these types of high-risk congregate settings, screening testing might complement diagnostic testing of symptomatic persons by identifying asymptomatic infected persons (*18**,**19*). When implemented, screening testing strategies should include all persons, irrespective of vaccination status. Screening testing might not be cost-effective in general community settings, especially if COVID-19 prevalence is low (*20**,**21*).

**Isolation.** Symptomatic or infected persons should isolate promptly, and infected persons should remain in isolation for ≥5 days and wear a well-fitting and high-quality mask or respirator if they must be around others. Infected persons may end isolation after 5 days, only when they are without a fever for ≥24 hours without the use of medication and all other symptoms have improved, and they should continue to wear a mask or respirator around others at home and in public through day 10^¶¶¶^ (Figure) (*22**,**23*). Persons who have access to antigen tests and who choose to use testing to determine when they can discontinue masking should wait to take the first test until at least day 6 and they are without a fever for ≥24 hours without the use of fever-reducing medication and all other symptoms have improved. Use of two antigen tests with ≥48 hours between tests provides more reliable information because of improved test sensitivity (*24*). Two consecutive test results must be negative for persons to discontinue masking. If either test result is positive, persons should continue to wear a mask around others and continue testing every 48 hours until they have two sequential negative results.****

**FIGURE:**
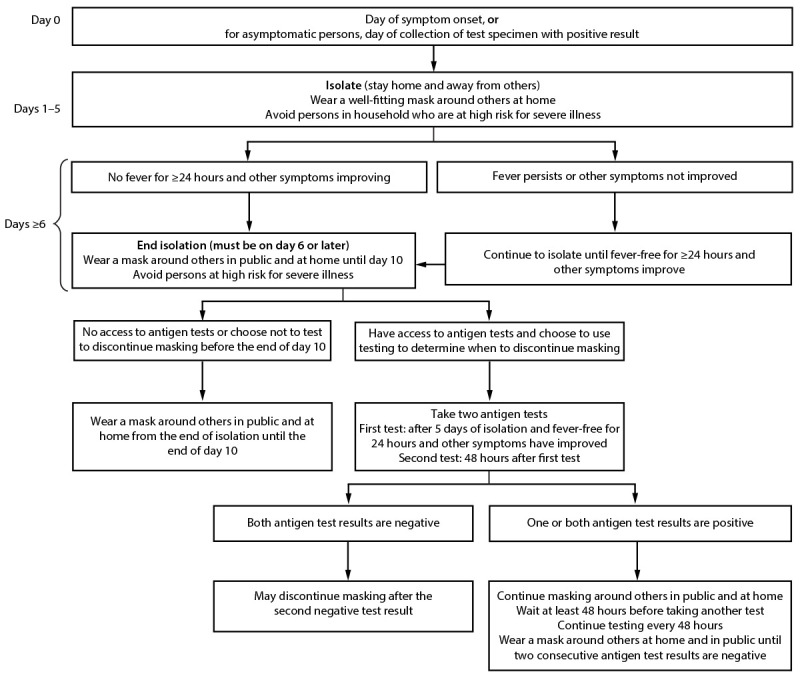
Recommendations for isolation,* masking,^†^ and additional precautions for persons with COVID-19 illness^§^ or who receive a positive SARS-CoV-2 test result^¶^^,^** — United States, August 2022 * Symptomatic persons should isolate immediately and get tested. They should remain in isolation until they receive a test result. If the test result is positive, they should follow the full isolation recommendations. Asymptomatic persons should begin counting isolation from the first full day after a positive test result (day 0 is the date the test specimen was collected). If an infected person develops symptoms after a positive test result, the isolation count starts again with day 0 being the first day of symptoms. ^†^ Persons at high risk for severe illness should wear a mask or respirator (N95/KN95) that provides more protection indoors in public at medium and high COVID-19 Community Levels. All persons should wear well-fitting masks or respirators indoors in public at high COVID-19 Community Levels. https://www.cdc.gov/coronavirus/2019-ncov/your-health/covid-by-county.html ^§^ Persons who had moderate illness from COVID-19, including those who show evidence of lower respiratory disease such as shortness of breath or difficulty breathing should isolate for ≥10 days. Persons who had severe illness from COVID-19, including those who were hospitalized and those who required intensive care or mechanical ventilation, and persons with immunocompromising conditions should isolate for ≥10 days and consult with a health care provider to determine end of isolation. https://www.covid19treatmentguidelines.nih.gov/overview/clinical-spectrum/ ^¶^ Infected persons can contact their health care provider to discuss their test results and available treatment options. They should monitor fever and other symptoms. If they develop an emergency warning sign, they should seek emergency medical care immediately. Emergency warning signs include trouble breathing; persistent pain or pressure in chest; new confusion; inability to awaken or stay awake; and pale, gray, or blue-colored skin, lips, or nailbeds, depending on skin tone. https://www.cdc.gov/coronavirus/2019-ncov/symptoms-testing/symptoms.html ** If symptoms worsen from the end of isolation through day 10, infected persons should restart isolation; they should consider consulting with a health care provider to determine care.

**Managing SARS-CoV-2 exposures.** CDC now recommends case investigation and contact tracing only in health care settings and certain high-risk congregate settings.^††††^ In all other circumstances, public health efforts can focus on case notification and provision of information and resources to exposed persons about access to testing. Persons who have had recent confirmed or suspected exposure to an infected person should wear a mask for 10 days around others when indoors in public and should receive testing ≥5 days after exposure (or sooner, if they are symptomatic), irrespective of their vaccination status.^§§§§^ In light of high population levels of anti–SARS-CoV-2 seroprevalence (*7*,*16*), and to limit social and economic impacts, quarantine of exposed persons is no longer recommended, regardless of vaccination status.

## Protecting Persons Most at Risk for Severe Illness

Multiple nonpharmaceutical and medical prevention measures are available to substantially reduce the risk for medically significant illness and death among persons at particularly high risk for these outcomes because of older age, disability, moderate or severe immunocompromise (*25*), or other underlying medical conditions (including pregnancy) (*26*). In addition to recommending that persons stay up to date with vaccination, public health strategies to protect persons at high risk include use of masks or respirators (i.e., specialized filtering masks such as N95/KN95s) that provide more protection for the wearer,^¶¶¶¶^ preexposure prophylaxis if indicated (e.g., for persons who are immunocompromised), and early access to and use of antivirals. At medium and high COVID-19 Community Levels, persons at high risk for severe illness and their contacts should consider wearing well-fitting masks or respirators that provide more protection to the wearer because of better filtration and fit to reduce exposure and infection risk. Persons who have household or social contact with persons at high risk should consider self-testing to detect infection before contact at medium and high COVID-19 Community Levels. Public health efforts should promote health equity by purposefully reaching out to all populations at high risk for severe illness to broaden access to preexposure prophylaxis, testing, and oral antivirals. Public health practitioners and organizations should consider the characteristics of their local or setting-specific populations when determining whether to strengthen or add prevention strategies that supplement disease control efforts and protect those persons at highest risk for severe illness or death. Strengthening public health communications and messaging can also help persons assess their personal level of risk for severe illness and use that knowledge to choose preventive behaviors to protect themselves and those around them.*****

## Discussion

COVID-19 remains an ongoing public health threat; however, high levels of vaccine- and infection-induced immunity and the availability of medical and nonpharmaceutical interventions have substantially reduced the risk for medically significant illness, hospitalization, and death from COVID-19. As transmission of SARS-CoV-2 continues, the current focus on reducing medically significant illness, death, and health care system strain are appropriate and achievable aims that are supported by the broad availability of the current suite of effective public health tools. Rapid identification of emergent variants necessitating a shift in prevention strategy makes continued detection, monitoring, and characterization of novel SARS-CoV-2 variants essential. Incorporating actions to mitigate the impact of COVID-19 into long-term sustainable routine practices is imperative for society and public health.

**Summary**

**What is already known about this topic?**

High levels of immunity and availability of effective COVID-19 prevention and management tools have reduced the risk for medically significant illness and death.

**What is added by this report?**

To prevent medically significant COVID-19 illness and death, persons must understand their risk, take steps to protect themselves and others with vaccines, therapeutics, and nonpharmaceutical interventions when needed, receive testing and wear masks when exposed, receive testing if symptomatic, and isolate for ≥5 days if infected.

**What are the implications for public health practice?**

Medically significant illness, death, and health care system strain can be reduced through vaccination and therapeutics to prevent severe illness, complemented by use of multiple prevention methods to reduce exposure risk and an emphasis on protecting persons at high risk for severe illness.

## Footnotes

*
https://www.cdc.gov/coronavirus/2019-ncov/vaccines/stay-up-to-date.html

† Vaccination is also effective in preventing multisystem inflammatory syndrome in children, a rare but severe postinfectious hyperinflammatory condition that can occur after mild or asymptomatic infection among children. https://www.cdc.gov/mmwr/volumes/71/wr/mm7102e1.htm

§
https://covid.cdc.gov/covid-data-tracker/#vaccinations_vacc-people-additional-dose-totalpop

¶ Adults and adolescents aged ≥12 years might be eligible for Evusheld, a combination of two monoclonal antibodies (tixagevimab copackaged with cilgavimab, administered as two consecutive intramuscular injections), if they are moderately or severely immunocompromised and might not mount an adequate immune response to COVID-19 vaccination or have a history of severe allergic reactions to COVID-19 vaccines, and do not currently have COVID-19 and have not recently had close contact with someone with COVID-19. https://www.cdc.gov/coronavirus/2019-ncov/need-extra-precautions/people-with-medical-conditions.html#preventive; https://www.fda.gov/media/154701/download

** Paxlovid, which is taken orally, and remdesivir, administered intravenously, are the current primary treatments, with Lagevrio and monoclonal antibodies as alternates (https://www.covid19treatmentguidelines.nih.gov/management/clinical-management/). Some patients who have completed a 5-day course of Paxlovid and have recovered can experience recurrent illness; patients experiencing COVID-19 rebound should be advised to follow CDC’s recommendations for isolation (https://emergency.cdc.gov/han/2022/pdf/CDC_HAN_467.pdf).

††
https://www.cdc.gov/coronavirus/2019-ncov/need-extra-precautions/people-with-medical-conditions.html

§§
https://aspr.hhs.gov/TestToTreat/Pages/default.aspx

¶¶
https://www.fda.gov/media/155049/download

*** CDC recommends the use of three indicators to measure COVID-19 Community Levels: 1) new COVID-19 hospital admissions per 100,000 population in the last 7 days; 2) percentage of staffed inpatient beds occupied by patients with confirmed COVID-19 (7-day average); and 3) new COVID-19 cases per 100,000 population in the last 7 days. The COVID-19 Community Level is determined by the higher of the new admissions and inpatient beds occupied metrics, based on the current level of new cases per 100,000 population in the last 7 days. The indicators combine to result in three COVID-19 Community Levels: low, medium, and high. COVID-19 Community Levels do not apply in health care settings, such as hospitals and nursing homes. Performance of COVID-19 Community Levels (including the component metrics and performance overall) will be reassessed and adjusted, if necessary, to accommodate changes in factors such as viral dynamics, emergence of novel variants of concern, or ecological changes that affect indicator data (e.g., shifts to greater use of self-testing or changes in reporting cadence).

††† Recommendations are additive, in that recommendations for the low community level apply to the medium and high levels, and the additional recommendations for medium level apply to the high level.

§§§ In high-risk settings such as nursing homes, modeling suggests that serial screening testing might be effective when performed very frequently (e.g., daily), although such high frequency is likely logistically challenging. https://academic.oup.com/cid/advance-article/doi/10.1093/cid/ciac505/6611848

¶¶¶ Persons at high risk of severe illness should wear masks or respirators (N95/KN95s) that provide more protection indoors in public at medium and high COVID-19 Community Levels. All persons should wear well-fitting masks or respirators indoors in public at high COVID-19 Community Levels (https://www.cdc.gov/coronavirus/2019-ncov/your-health/covid-by-county.html). Persons who had moderate illness from COVID-19, including those who show evidence of lower respiratory illness such as shortness of breath or difficulty breathing, should isolate for ≥10 days. Persons who had severe illness from COVID-19, including those who were hospitalized and those who required intensive care or mechanical ventilation, and persons with immunocompromising conditions should isolate for ≥10 days and talk with a health care provider to determine end of isolation. https://www.covid19treatmentguidelines.nih.gov/overview/clinical-spectrum/

**** Persons who choose to use testing to determine when to discontinue masking can end isolation after day 5 even if they receive a positive test result. They should continue wearing a well-fitting and high-quality mask around others at home and in public until they receive two consecutive negative test results, with tests taken ≥48 hours apart. For some persons, this might mean that they will continue masking longer than 10 days since symptom onset. https://www.fda.gov/medical-devices/coronavirus-disease-2019-covid-19-emergency-use-authorizations-medical-devices/in-vitro-diagnostics-euas-antigen-diagnostic-tests-sars-cov-2

†††† Case investigation and contact tracing are fundamental activities that involve working with a patient (symptomatic or asymptomatic) who has received a diagnosis of an infectious disease to identify and provide support to persons (contacts) who might have been infected through exposure to the patient. CDC recommends that health departments prioritize case investigation and contact tracing in high-risk congregate settings, for clusters or outbreaks that involve unusual clusters of cases, or for novel or emerging variants that might pose significant risks for severe illness, hospitalization, or death. https://www.cdc.gov/coronavirus/2019-ncov/php/contact-tracing/contact-tracing-plan/prioritization.html

§§§§ For persons unable to wear a mask or children aged <2 years, other prevention actions should be taken, such as additional physical distancing and increased ventilation. Exposed persons who develop symptoms should receive testing promptly.

¶¶¶¶ Masks and respirators can provide different levels of protection depending on the type of mask and how they are used. Loosely woven cloth products provide the least protection, layered finely woven products offer more protection, well-fitting disposable surgical masks and KN95s offer even more protection, and well-fitting CDC National Institute for Occupational Safety and Health–approved respirators (including N95s) offer the highest level of protection. https://www.cdc.gov/coronavirus/2019-ncov/prevent-getting-sick/types-of-masks.html#DifferentSituations

*****
https://www.cdc.gov/coronavirus/2019-ncov/your-health/factors-affecting-risk-of-getting-sick.html
